# Exploration of chaos game representation and integrative deep learning approaches for whole-genome sequencing-based grapevine genetic testing

**DOI:** 10.1093/bioadv/vbaf193

**Published:** 2025-09-01

**Authors:** Andrew Vu, Brendan Park, Yifeng Li, Ping Liang

**Affiliations:** Department of Computer Science, Brock University, St. Catharines, ON L2S 3A1, Canada; Department of Computer Science, Brock University, St. Catharines, ON L2S 3A1, Canada; Department of Computer Science, Brock University, St. Catharines, ON L2S 3A1, Canada; Department of Biological Sciences, Brock University, St. Catharines, ON L2S 3A1, Canada; Centre for Biotechnology, Brock University, St. Catharines, ON L2S 3A1, Canada; Department of Biological Sciences, Brock University, St. Catharines, ON L2S 3A1, Canada; Centre for Biotechnology, Brock University, St. Catharines, ON L2S 3A1, Canada

## Abstract

**Motivation:**

The identification of grapevine species, cultivars, and clones associated with desired traits is an important component of viticulture. True-to-type identification is very challenging for grapevine due to the existence of a large number of cultivars and clones and the historical issues of synonyms and homonyms. DNA-based identification, superior to morphology-based methods, has been used as the current standard true-to-type method for grapevine, but not without shortcomings, such as the limited number of biomarkers and accessibility of services.

**Results:**

To overcome some of the limitations of traditional microsatellite-marker-based genetic testing, we explored a whole-genome-sequencing (WGS)-based approach to achieve the best accuracy at an affordable cost. To address the challenges of the extreme high dimensionality of the WGS data, we examined the effectiveness of using chaos game representation (CGR) to represent the genome sequence data and using deep learning for species and cultivar identification. CGR images provide a meaningful way to capture patterns for use with visual analysis, with the best results showing a 99% balanced accuracy in classifying five species, and a 80% balanced accuracy in predicting 41 cultivars. Our preliminary research highlights the potential for CGR and deep learning as a complementary tool for WGS-based species- and cultivar-level classification.

**Availability and implementation:**

Our implementation, including the pipeline for data processing and the four predictive models, is available at https://github.com/pliang64/CGR.

## 1 Introduction

Genetic testing for crops, such as grapevine, has become increasingly important in modern agriculture. This is because it allows for the identification of genetic variants associated with desired traits such as disease resistance, crop yield, and crop quality (Schuster 2008). Genetic testing for accurately identifying grape materials in vineyards is vital but challenging, due to the existence of many species and their hybrids and a vast number of *Vitis vinifera* cultivars and clones, further compounded by the long historical use of confusing and erroneous synonyms and homonyms. Old and visual methods based on leaf or grape characteristics have proven to be of very limited use, and the fields of genetic testing for humans and other species have long moved to the use of DNA-based methods, which employ a specific type of DNA sequences called simple short repeats (SSR) or short tandem repeats (STRs) that show a high level of inter- and intra-species variations (Thomas and Scott 1993, Bowers and Meredith 1997). While effective in general, STR-based DNA testing for grapevine has limitations in use with some hybrids and in differentiating clones due to the use of only a handful of markers relying on variation in length rather in sequence. Furthermore, accessibility to such services is limited to a few places around the globe. As a result, users from many countries, including Canada and most other countries, need to send samples across international borders, incurring logistic hurdles, high expenses, and delays. Therefore, there is a strong need for more robust, accessible, and cost-effective genetic testing methods. The advent of next-generation sequencing (NGS) technologies has enabled the generation of a large amount of DNA sequence data quickly, making the very desirable but once cost prohibitive whole-genome sequencing (WGS) affordable (van Dijk *et al.* 2014, Kunej *et al.* 2020). In the last two decades, thanks to NGS, many significant advances have been made in grape genetics, including the development of high-quality grape reference genomes and the accumulation of WGS data for thousands of grape samples, covering most grape species, cultivars, and clones around the world (Canaguier *et al.* 2017, Liang *et al.* 2019, Dong *et al.* 2023, Shi *et al.* 2023, Urra *et al.* 2023).

There have been a few recent studies exploring the feasibility of using NGS-based approaches for genotyping STR markers for cultivars (Vondras *et al.* 2019, Kunej *et al.* 2020) and for identifying markers to differentiate clones for a few elite cultivars through WGS. These studies, despite being limited in number and scope, clearly demonstrate the existence of sufficient genetic differences even among clones of grapevines. This has motivated us to explore a direct WGS-based approach, which can potentially provide the complete set of genomic variants across species, cultivars, and clones of grapevine, to address the shortfalls in current genetic testing for the crop.

Although highly promising, the WGS-based genetic testing faces new challenges, in particular the complication in computational analysis of a large amount of raw DNA sequence data (> 10 GB/sample). This research was designed specifically to address the efficiency of the computational data process in WGS-based genetic testing by exploring the use of computational methods, such as chaos game representation (CGR) (Jeffrey 1990) to visualize sequence data, and machine learning algorithms in pattern recognition. By tackling these issues, the study seeks to enhance the robustness and accessibility of grapevine genetic testing, contributing to the broader field of agricultural genomics and shedding further insight into areas that have had scarce research, particularly concerning the use of CGR in biological data. This research has two major contributions. (1) Development of a novel CGR-based methodology using deep learning models: We introduce a new approach using CGR for genomic data representation, specifically for grapevine genetic analysis. This method offers an alternative way to handle the high-dimensional nature of both chromosomal- and genome-level sequence data. We creatively explored four methods (chromosome-wise, early-integration, late-integration, and whole-genome) to integrate CGR with established deep learning models to perform visual analysis of the genomic data. This integration significantly improves the efficiency in processing WGS data compared to sequence similarity-based approaches. This paper outlines a novel approach to address the limitations of the current STR-based method in cost efficiency and availability. This contribution may be valuable to users in regions (e.g. Canada) with limited access to traditional genetic testing service. Our goal with this research is to provide a more cost-effective and readily available WGS-based method for genetic testing. (2) Development of a specialized bioinformatic pipeline and database: We created a bioinformatic pipeline and a comprehensive database specifically designed for grapevine genetic testing. This system streamlines the analysis and genetic testing process. While our research focused on grapevines as a model, this methodology has the potential for broader applications in genetic testing for other species. Furthermore, the framework of combining CGR and machine learning for pattern identification may have broader applications in genomic research.

## 2 Related work

### 2.1 Deep learning

Residual Network (ResNet) was introduced in 2015 (He *et al.* 2016) as an improvement over convolutional neural networks (CNNs) (LeCun *et al.* 1989), demonstrating a shift in how deep neural networks are designed. Deeper neural networks were difficult to train, and ResNet presents a novel architecture that allows the training of networks that are substantially deeper than prior works. The key innovation of ResNet is the introduction of residual blocks which are used to skip connections, or shortcuts, to jump over some layers. This addresses a degradation problem that occurs as network depth increases. The skip connections allow networks to propagate signals across many layers without attenuation, making it easier to train deeper networks, mitigate issues of vanishing gradients, and help preserve the original information that can be lost in deep networks. Vanishing gradients refer to problems in the training of neural networks that occur during back-propagation. Errors from the output layers are propagated back through the network to adjust the weights of each layer, which involves calculating the gradients of the error relative to the weight. Vanishing gradients refer to gradients becoming small such that the earlier layers are no longer adjusted. Alternative functions such as Rectified Linear Unit (ReLU) attempt to address this issue and have demonstrated improved results on generative stochastic neural networks, in particular, restricted Boltzmann machines (RBMs) (Nair and Hinton 2010). ResNet alleviates this issue through architectural changes, allowing mapping block to be connected to the fully connected layer. ResNets have been demonstrated to be easier to optimize and capable of gaining accuracy from considerably increased depth, resulting in top performance in competitions after the initial proposal (He *et al.* 2016).

Vision Transformers (ViTs) attempt to adapt Transformer architectures, an attention model typically used in natural language processing, for application with images. This is a significant departure, demonstrating that convolution is not necessary for computer vision and that pure Transformers can be applied directly to image patches to perform classification tasks (Dosovitskiy *et al.* 2021). In comparison, CNNs have a greater image-specific inductive bias in that the locality, two-dimensional neighbourhood structure, and translation equivariance are baked into each layer of the model, whereas ViT has self-attention layers that are global (Dosovitskiy *et al.* 2021). This indicates that there is greater flexibility in representing relationships and capturing global context, since long-range dependencies can be captured in an image. The Transformer architecture relies on attention mechanisms, such as self-attention to draw global dependencies among input tokens and cross-attention between input and output, as opposed to recurrence and convolutions, which capture local dependencies. Attention functions compute an output as a weighted sum of values, where the weights are determined by the compatibility of queries with a set of key-value pairs (Vaswani *et al.* 2017). Transformers are typically used for computational linguistic tasks. ViT separates images into sequences of two-dimensional patches, rather than a grid of pixels like in CNNs. This treats patches as words or tokens in a sentence. The image is then separated into fixed-size patches, flattened to a one-dimensional array used as embedding for the Transformer encoder. The patch is used to create the query, key, and value for the attention mechanism (Dosovitskiy *et al.* 2021). This process also creates a limitation in applying ViTs to high-resolution images, such as CGRs, since the number of tokens scales quadratically with the resolution.

### 2.2 Deep learning in grapevine applications

Existing work applying deep learning principles in the field of grapevine cultivar identification exists, particularly from the computer vision perspective. In 2021, a proof-of-concept study demonstrated the use of leaf imaging and CNNs for the identification of Iranian varieties (Nasiri *et al.* 2021). High accuracy was obtained using the VGG16 architecture for use as a complementary tool to ampelography and quantitative genetics by relying on leaf imaging using representative true-to-type images of leaves from six grapevine cultivars in Iran (Nasiri *et al.* 2021). Their experiments succeed in applying a CNN framework to achieve automatic identification of grapevine cultivars, demonstrating the potential application of visual models in the field of agriculture and plant sciences, particularly for tasks such as cultivar identification or classification. Furthermore, it highlights how advanced image processing techniques can be utilized for detailed and accurate plant identification (Nasiri *et al.* 2021). Despite this, the use of visual analysis for an ampelographic approach is still limited, since the distinction between morpholographic characteristics of leaf-based images can be very subtle if any among clones and some closely related cultivars and can be subject to changes in growing conditions. To overcome such limitations, our research investigates alternative uses of visual models using sequence-based methods to encompass a broader spectrum of grapevine species and cultivars. One-dimensional sequence modelling methods, such as recurrent neural networks and the vanilla Transformer, are unsuitable for genomic sequence modelling due to the extreme length (in magnitude of millions).

### 2.3 Chaos game representation

Genomic sequences are analysed to identify lineage-specific patterns and motifs for classification, but this process often requires significant computational resources. As an alternative approach, the implementation of a CGR technique on DNA was pioneered by Jeffrey in 1990 (Jeffrey 1990) where a square representation was used instead of a triangle as the basis for CGR. This maps DNA sequences onto a two-dimensional plane, where the four vertices of the square represent the four nucleotides, adenine (A), cytosine (C), guanine (G), and thymine (T). This method generates distinctive patterns, highlighting DNA sequence features, such as structural variations and sequence repeats. Notably, random sequences did not exhibit these distinctive patterns which emerged when applied to DNA sequences (Löchel and Heider 2021). By reducing complex genomic data into visual patterns, CGR has the potential to offer insightful views into the structural and evolutionary characteristics of species, making it particularly useful in distinguishing grapevine species and cultivars. As of this writing, to best our knowledge, there is no work applying CGR representation of whole-genome sequences for any species, and thus it is unknown what the representation looks like and whether it is informative to help distinguish grapevine species and cultivars.

## 3 Methods

The exploration of agricultural science through computational techniques, bioinformatics, and machine learning is exemplified in this paper through the application of CGR to visualize extremely long genome sequences for classification of grapevine species and cultivars. A Python-based tool has been developed to generate CGR images from grapevine genome sequence data, simplifying the visualization of the data and speeding up the downstream analysis to enable the potential for distinguishing grapevine varieties. This study investigates the use of deep image models to classify grapevine species and cultivars based on these CGR images, testing the method’s capacity to efficiently process large volumes of genomic data. The innovative synergy of CGR and deep image models presents a novel approach in grapevine species and cultivar identification for complementing or even replacing traditional grapevine true-to-type methods.

### 3.1 Generation of DNA sequences representing genome-wide variants of grapevine samples

WGS data for a total of 4312 grapevine samples were retrieved from the NCBI SRA (https://www.ncbi.nlm.nih.gov/sra) and CNCB GSA (https://ngdc.cncb.ac.cn/gsa) databases in compressed FASTQ format (fastq.gz) (Katz *et al.* 2021). These samples cover 46 species in the genus of *Vitis* and 4 cross-species hybrids, among which over 4100 samples belong to *Vitis vinifera*, which is the most commonly used grape species with more than 3200 cultivars and clones. These WGS data were all generated using the Illumina platforms as pair-end reads and with genome coverage ranging from 10 to 100 times. They were then used to generate a list of genome-wide variants covering single-nucleotide polymorphisms (SNPs) and small insertions and deletions (indels) using an in-house pipeline. The process involves the use of BWA (mem) (Li and Durbin 2009) for aligning the WGS reads to the reference genome (NCBI acc#: GCF_000003745.3) and use of bcftools (Li 2011) for variant calling (with the -v option disabled in the ‘bcftools call’ step to ensure all samples have identical number of loci in the variant lists). Variants in binary variant call format (bcf) were then converted to continuous pseudo DNA sequences in FASTA format with the base order dictating their positions in the chromosomes and the bases at the same location corresponding to orthologous position across samples. Sequences were presented in individual chromosomes (i.e. one sequence per chromosome per sample) and also as whole genomes by concatenating individual chromosome sequences as one sequence per sample. The total length of DNA sequences for individual chromosomes ranges from 4.5Mbp to 8.1Mbp and is 112Mbp for the whole genome. While the detection of variants used all these 4312 samples, only those with at least three samples per species or cultivar class were used, as this is the minimal number of samples required for training, validation, and test.

### 3.2 Visualizing sequence variations in grapevine genomes with CGR

**Figure 1.**
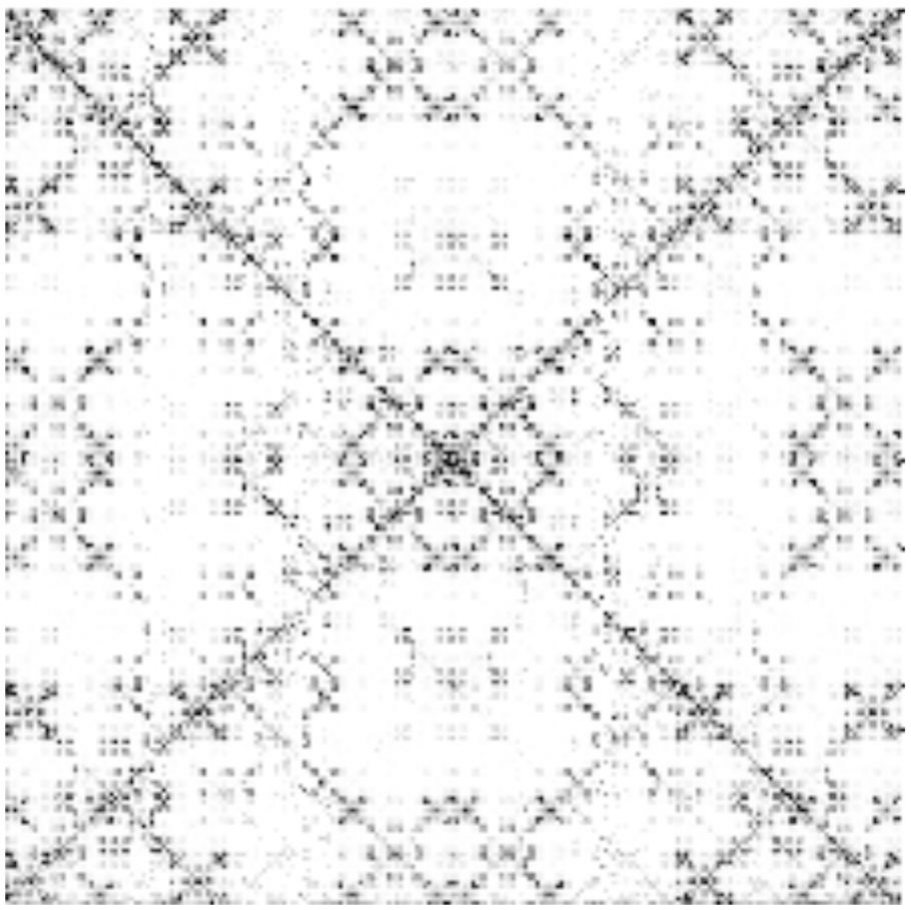
An example for a normalized CGR image sequence representing variants in chromosome 1 of the grapevine sample (ID: SRR5891911).

DNA sequences stored in FASTA format files were parsed using the BioPython library to extract each record as a separate record (Cock *et al.* 2009), with the CGR coordinates calculated for a given DNA sequence. Specifically, a specific mapping algorithm assigns the nucleotides ‘A’, ‘C’, ‘G’, and ‘T’ to each corner of a two-dimensional plane ranging from 0 to 1 (Jeffrey 1990) with ‘A’ corresponding to coordinate (0,0), ‘C’ to (0,1), ‘G’ to (1,1), and ‘T’ to (1,0). By always placing a pixel point at the midpoint between the prior point and the corner of the current base, it converts a 1D DNA sequence into a 2D image. Any non-standard or missing nucleotides in the sequence default to a midpoint mapping, ensuring a consistent and comprehensive representation. Normalization is also applied before generating the final image based on the matrix, such as in Fig. 1.

#### 3.2.1 Preprocessing of CGR data from large datasets of DNA sequences

To enhance the accuracy and generalization capabilities of CNNs, a robust processing of large genomic sequence datasets is vital. Recognizing this, we created an automated and streamlined approach to convert the sequence data into CGR images in conjunction with additional categorization modules to efficiently handle inputs and outputs through command line interface flags. This resulted in a system that manages file searching and processing logic and also ensures the organized categorization of CGR output images based on species, chromosomes, and other specified parameters. This approach allows for the accommodation of large FASTA files and produces structured, pre-processed datasets, pivotal for downstream in-depth analysis and model training.

The CGR data were organized by species and vinifera cultivars, with chromosomal and whole genome datasets for each sample. For both species and cultivars, the available CGR data were divided into representative training, validation, and test sets. This distribution ensures a systematic approach to data segregation, setting the stage for training and fine-tuning visual models on sequence data. A full breakdown of the final dataset partitioning can be found in the supplementary data file (Tables S1 and S2, available as supplementary data at *Bioinformatics Advances* online). Briefly, for species classifications, we used a total of 297 CGRs from pseudo-DNA sequences representing the genotype data for a total of 19 million variation loci. These were divided into three sets of 198, 40, and 59 for training, validation, and test, respectively. Collectively, these CGRs cover 19 class of species, among which 3 are hybrids, while the remaining 16 are for individual species. The group size ranged from 1 to 42 for the training set, 1 to 7 for the validation set, and 1 to 11 for the test set. For cultivar classification, we used a total of 668 CGRs, all for cultivars of *Vitis vinifera*, which is the dominant species used worldwide. The CGRs were divided into sets of 326, 167, and 175 for training, validation, and test, respectively. Collectively, these CGRs cover a total of 164 cultivars with group size ranging from 1 to 7, 1 to 2, and 1 to 2 for training, validation, and test, respectively.

#### 3.2.2 Fine-tuning of pretrained models on CGR data

Fine-tuning is an approach in machine learning that makes use of pretrained models, which are then adjusted to suit a specific task or dataset through additional training. Utilizing pretrained models yields benefits including less computing time compared to training a model from scratch and overcoming data scarcity. In our work, we performed fine-tuning, evaluation, and classification using pretrained models on the preprocessed CGR images as further described in following sections.

Class weight calculation is necessary in our experiments, as some species and cultivars of grapevine were overrepresented relative to others, leading to imbalanced data, in particular *Vitis vinifera* for species classification. Class weights are used in the loss function to mitigate biases towards overrepresented classes by assigning more weight to less frequent classes and giving more attention to these classes during training time. Our approach for balancing multiple classes is based on the inverse class frequency and is extended from the binary-class focal loss originally for image segmentation (Lin *et al.* 2017).

We calculate the weight for each class inversely proportional to its frequency in the training dataset. Specifically, let $n_{c}$ be the number of samples of class *c*, *C* be the total number of unique classes, and *N* be the total number of samples across all classes, that is $N=\sum_{c=1}^{C}n_{c}$. The class weight for each class is calculated as:


(1) $$w_{c}=\frac{N}{C\times n_{c}}$$

where $w_{c}$ is the weight for class *c*. By calculating the weight for a class *c* as the total number of samples *N* over *C* times $n_{c}$, the classes with a lower frequency have a higher weight in the loss function. This gives more importance to minority classes during model training. The objective for a classification task with classes in the range [1, *C*] is calculated using the weighted cross-entropy loss function. This function computes the loss for each sample in a minibatch and then averages these losses. The averaged loss over a minibatch of size *M* is given by:


(2) $$\ell (x,y)=\frac{1}{M}\sum_{m=1}^{M}l_{m},$$

where each individual loss $l_{m}$ for a sample in the minibatch is calculated as:


(3) $$l_{m}=-w_{y_{m}}\;\text{log}\;\frac{\;\text{exp}(x_{m,y_{m}})}{\sum_{c=1}^{C}\;\text{exp}\;(x_{m,c})}.$$

Here, $x_{m}$ represents the input logits of the *m*-th sample, $y_{m}$ is the corresponding actual target label, and $w_{y_{m}}$ is its class weight. This weighted loss function ensures that it is averaged over all samples in the minibatch, making the loss less sensitive to batch size (Lin *et al.* 2017).

Hyperparameters and external settings for model training were used, including standard hyperparameters such as batch size, learning rate, and epochs, with a random seed to enable reproducibility. The pretrained models, including ResNet and ViT, were obtained from the PyTorch Torchvision package (Marcel and Rodriguez 2010), offering the ability to load pretrained model parameters and tailor the final layers to the dataset’s class count to enhance model adaptability for our CGR image analysis. Our framework was designed to be generic with the capacity for a variety of deep image models to be utilized. Our experiments were primarily conducted using ResNet due to its well-known performance, flexibility in variable image resolutions, and as a result of initial preliminary experiments using the chromosome-wise method to examine the feasibility of the process.

#### 3.2.3 Fine-tuning and integration methodology

**Figure 2.**
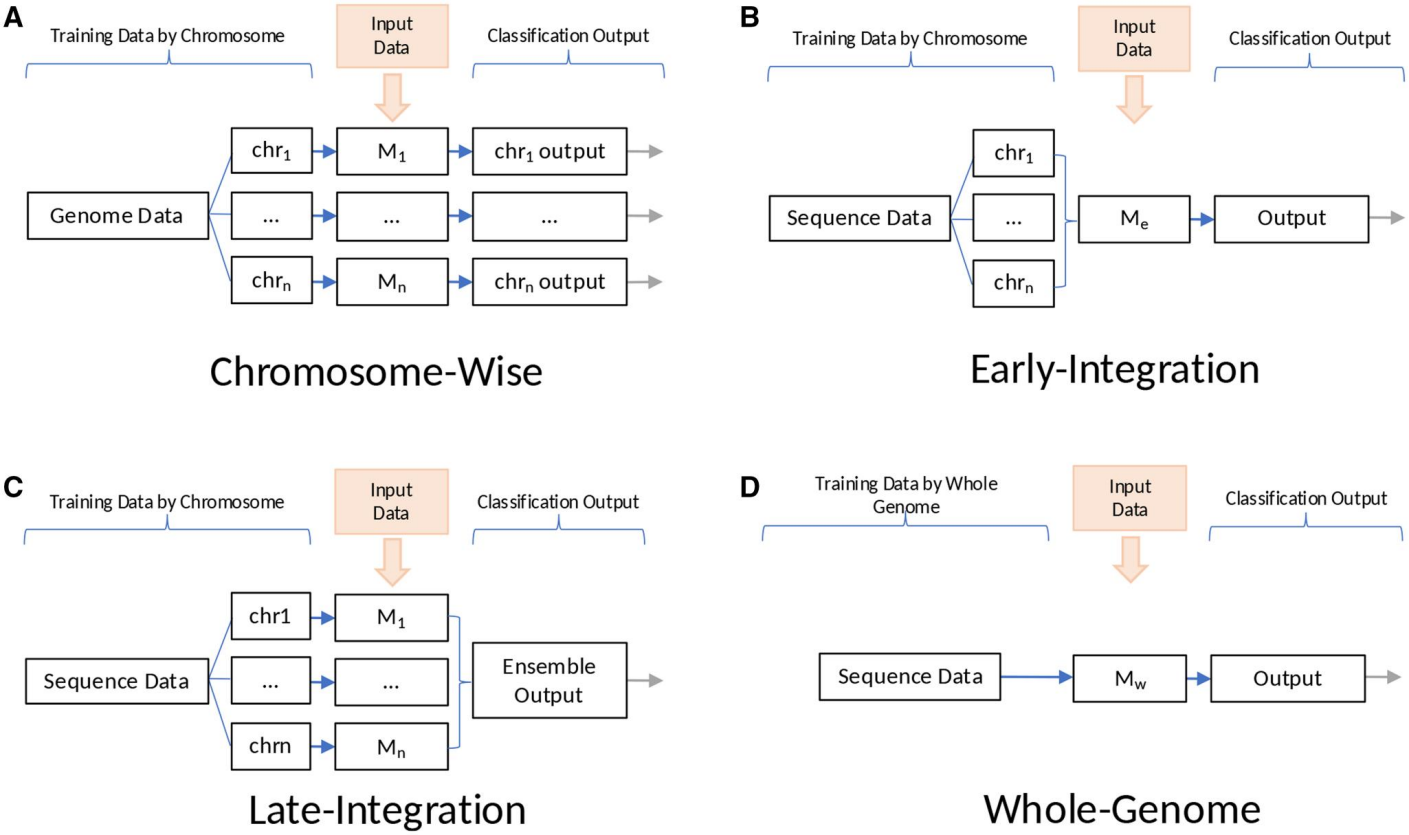
Illustration of our fine-tuning and integration methods. (A) Chromosome-wise method. (B) Early-integration method. (C) Late-integration method. (D) Whole-genome method. M: deep image model.

We tested a novel multifaceted approach for fine-tuning and evaluating models, particularly focusing on chromosome-specific variations and whole-genome analysis. The methodologies are illustrated in Fig. 2 and explained as follows:

1. Chromosome-wise method: As illustrated in Fig. 2A, this method involves fine-tuning individual models for each chromosome, with each model being trained and evaluated on data corresponding to its specific chromosome. The performance of these models is assessed independently using selected metrics. The average performance across all chromosome models is then computed, providing a comprehensive view of the model performance at the chromosome level.
2. Early-integration method: As shown in Fig. 2B, this approach encompasses data from all CGR datasets for each of 19 chromosomes to train a *single*, comprehensive model. In this way, a model can see the diverse CGRs of all individual chromosomes of the same sample without increasing the model size, resulting in a model that captures a more holistic representation of genomic data.
3. Late-integration method: This method employs a voting system, in which each of 19 chromosome-specific models casts a ‘vote’ based on its prediction and the final output is determined through a majority vote (see Fig. 2C). This ensemble technique underscores the collective intelligence of individual models (i.e. the *wisdom of the crowd* principle) (Li *et al.* 2018), potentially enhancing the predictive accuracy and robustness by aggregating diverse perspectives from each chromosome-specific model.
4. Whole-genome method: Diverging from the chromosome-centric methods, this approach utilizes the entire genome variant sequence of a sample to generate a single CGR. A model is trained on the genome-level CGRs, rather than training on CGRs for individual chromosome sequences, aiming to discern patterns that are visualized based on all chromosomes at the same time (see Fig. 2D).

Aside from species classification, this approach was also applied to the classification of cultivars of *Vitis vinifera* to evaluate its performance on identification of cultivars, which have much less sequence variation across samples.

#### 3.2.4 Evaluation metrics

We make use of balanced accuracy as a means of evaluating the models in our experiments. In imbalanced datasets, average accuracy may lead to false conclusions about models, particularly in cases of extreme imbalance. Given an imbalanced dataset, a model favouring classifying an overwhelmingly imbalanced class may achieve high levels of accuracy without meaningfully achieving generalizability (Brodersen *et al.* 2010).

Balanced accuracy calculates the average recall obtained on each class, giving equal weight to classes regardless of the class size (Pedregosa *et al.* 2011). This allows a model to be assessed on its true ability to correctly predict classes of unseen data (Brodersen *et al.* 2010). Denoting the number of true positives as TP, and false negatives as FN, for a given class *c* the recall *R* (class-wised accuracy) is given by:


(4) $$R_{c}=\frac{{\text{TP}}_{c}}{{\text{TP}}_{c}+{\text{FN}}_{c}}.$$

Then, the balanced accuracy for *C* classes is calculated as:


(5) $$BA=\frac{1}{C}\sum_{c=1}^{C}R_{c}.$$

This accounts for the recall of each class, preventing a majority class from dominating the metric and providing more importance for underrepresented classes.

## 4 Results

Experiments for both species-level and cultivar-level classification were performed by running 20 repetitions for each experimental setting. These experiments consist of exhaustive combinations of hyperparameters concerning CGR generation, predictive model integration, and model evaluations in terms of balanced accuracy. This section identifies and discusses the impact of these settings on the final model performance to empirically determine the impact on CGRs and evaluate their feasibility for use with deep learning frameworks. The full results of all experiments are provided in the supplementary file.

### 4.1 Visualizing sequence variations in grapevine genomes with CGR

Initial experiments with CGR were aimed to assess the feasibility and effectiveness of using CGR images for visualizing grapevine cultivar DNA sequences. Attempts to manage outliers through normalization techniques such as linear and logarithmic (log) normalization proved to be insufficient, as they led to significant detail loss, obscuring the DNA sequence information. To address these challenges, greyscale CGR images were introduced, providing a more nuanced visualization by incrementing greyscale values at each coordinate, a method that efficiently represented longer sequences and accounted for outliers without data distortion. Robust Scaler normalization (Pedregosa *et al.* 2011) was identified as an effective method for standardizing these images, preparing a comprehensive set of > 4000 samples, from which a subset was used for robust model training with careful consideration given to class balance and dataset size. We also examined the impact of image size, finding that size higher over 1024×1024 pixels did not yield any significant impact.

Subsequently, a series of deep vision models were trained on the aforementioned structured data to explore various architectures and integration strategies. ResNet18 was initially used for evaluating individual chromosomes, followed by experiments with the early-integration strategy (combining all 19 chromosomes) and the late-integration strategy with consensus voting for classification (separate models for each chromosome followed by majority vote). The chromosome-specific models showed notably better performance in terms of balanced accuracy than the integrated models. Further exploration with ResNet101, VGG11_bn, and ViT_b_16 indicated that, while ViT showed promising results, ResNet18 emerged as the most effective model in these initial tests. These experiments provided a foundational understanding of CGR’s potential in species classification, guiding further in-depth experimentation with various hyperparameter settings for comprehensive empirical analysis.

### 4.2 Overall impact of CGR hyperparameters on classification

We observed challenges regarding the handling of extreme outliers in mapping grapevine sequences to a two-dimensional plane. In addition to the use of normalization methods mentioned previously, CGR image resolution emerged as a crucial factor, with baseline resolutions set at 224×224, 512×512, and 1024×1024 pixels based on computational feasibility to optimize the dimensionality for model training. Class imbalance prevalent in species-based datasets was addressed by defining ‘very imbalanced’ (up to 60 training samples per class) and ‘mildly imbalanced’ (up to 15 training samples class) datasets, setting sample class limits, and examining the impact of class size on model performance across different proportions of the dataset. Extensive experiments with various CGR hyperparameters were conducted, utilizing a set of empirically derived hyperparameters: a batch size of 16, 200 training epochs, and a learning rate of 0.01 to ensure robust model convergence and optimal performance. This comprehensive analysis produced a significant volume of experiments, that is, 2520 for species classification and 1440 for cultivar classification, across multiple runs (20) to ensure reliability and mitigate randomness.

The supplementary file (Tables S3 and S4, available as supplementary data at *Bioinformatics Advances* online) contains all mean results from these experiments. Overall, the late-integration approach, which synthesizes information from various sources while preserving chromosomal differences, notably outperformed other methods. Moreover, the increase in model performance with larger class sizes emphasized the necessity of substantial class representation for the models to effectively learn and distinguish class features, underscoring the importance of class size and data representation in achieving accurate classification results. There was a dramatic increase in performance as the class size reached top 25% by class size. This observation underscores the importance of having a sufficiently large representation of each class in the dataset. Smaller class sizes might not provide the model with enough information to learn the distinguishing features of each class effectively, leading to poorer performance. Conversely, when examining the top 25% of classes in terms of available samples, performance dramatically increased. This trend was particularly evident in our study, suggesting that class size is a critical factor in successful application of machine learning models for classification tasks. Task-specific results are discussed below.

### 4.3 Species-level classification results

To fine-tune models for species-level classification and *Vitis vinifera* cultivar classification, separate CGR image datasets were prepared. The species dataset initially included 49 species but was refined by imposing a minimum of three samples per species and capping each at sixty samples to ensure sufficient data for training, validation, and evaluation, leaving us with 19 classes in the data (see Table S1 in the supplementary file, available as supplementary data at *Bioinformatics Advances* online). Experiments to classify grapevine species using CGR images explored the influence of image dimensions (224×224, 512×512, and 1024×1024), data normalization, class imbalancement, and various integration methods on classification performance.

**Figure 3.**
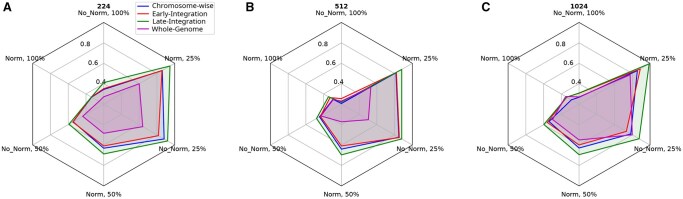
Comparison of integration methods for grapevine species classification. (A) 224×224. (B) 512×512. (C) 1024×1024. Base classifier: ResNet. Mean balanced accuracy on the test set over 20 training runs of each method and configuration is used.

A comparison of the top mean balanced accuracy across methods can be seen in Fig. 3, and the results illustrate complex relations between image dimension, class proportionality, normalization, and method used in species-level classification of grapevine samples. The late-integration method was shown to exhibit strong performance across various combinations of CGR hyperparameters, particularly at lower percentages of class proportionality.

**Figure 4.**
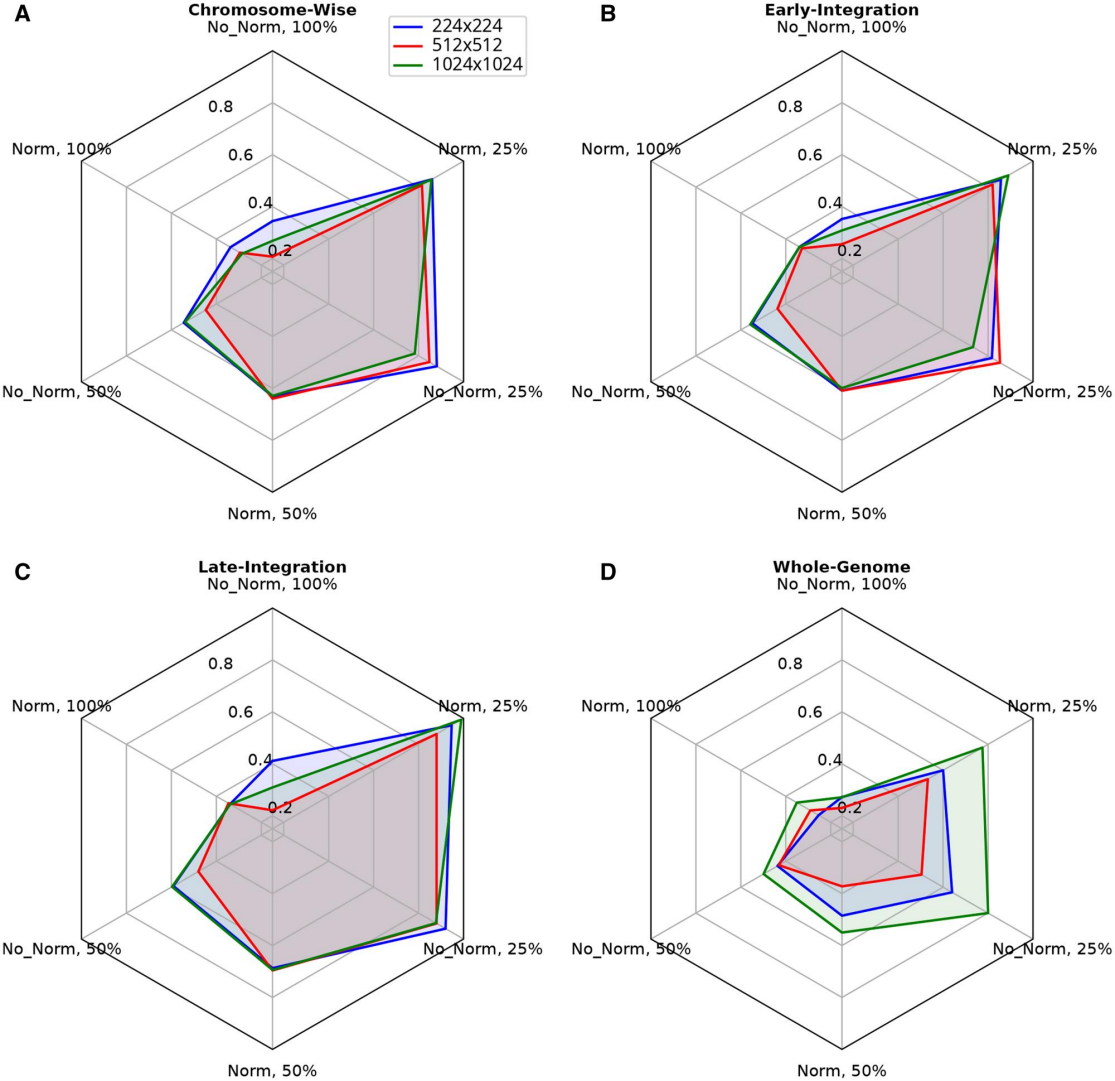
Comparison of CGR resolutions for grapevine species classification. (A) Chromosome-wise method. (B) Early-integration method. (C) Late-integration method. (D) Whole-genome method. Base classifier: ResNet. Mean balanced accuracy on the test set over 20 training runs of each method and configuration are shown.

Figure 4 shows resolution-wise comparison as an alternative visualization of the same results as in Fig. 3, with 1024×1024 shown to yield better results than 224×224 images based on the top mean balanced accuracy for each class proportion. This increase in accuracy varies, however, with no such increase found when comparing 512×512 images to 224×224.

An increase in performance given lower class proportionality with larger class size is expected, as a lower class proportionality indicates a simpler task. In our experiments, the optimal results with 25% of classes yielded a mean balanced accuracy of 99±2.4%, far greater than random chance. This can be explained in part by the disparity in the number of samples for each class available. Some species, such as *Vitis vinifera*, have a far greater number of samples available in our training data.

Other factors, such as normalization and class imbalancement, also appear to have an impact on performance, but this appears to vary given different combinations of hyperparameters, particularly at higher class proportionality. Overall, the classification performance of all models consistently exceeded the accuracy levels that would be anticipated if classifications were made at random, indicating that the models are capturing meaningful patterns in the CGRs of sequence data, rather than simply guessing. This suggests that CGR may be a useful complementary tool in genetic testing at the species level.

### 4.4 Cultivar-level classification results

In our experimentation with cultivar-level classification of grapevine samples, we saw a significantly greater number of classes relative to species-level classification. The cultivars examined all belonged to the species of *Vitis vinifera* and the training data comprised 164 classes (see Table S2 in the supplementary file, available as supplementary data at *Bioinformatics Advances* online).

Similarly to species-level classification, cultivar-level classification experiments extensively explored the dimensionality (224×224, 512×512, and 1024×1024), normalization, predictive methods, and class proportionality. Each setting was run 20 times using different random seeds. Class imbalance was not observed in cultivar-level classification, because the difference in class sizes did not vary significantly between classes.

**Figure 5.**
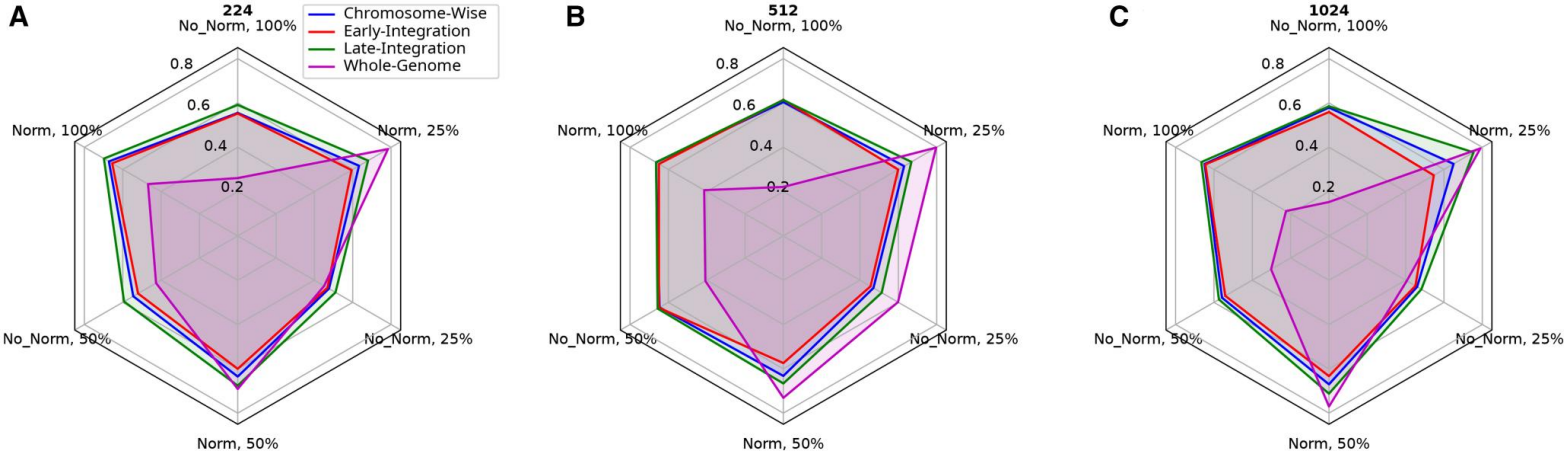
Comparison of integration methods for *Vitis vinifera* cultivar classification. (A) 224×224. (B) 512×512. (C) 1024×1024. Base classifier: ResNet. Mean balanced accuracy on the test set over 20 training runs of each method and configuration is shown.

As seen in Fig. 5, late-integration excelled at 100% class proportion across all dimensions, achieving the highest mean accuracy with 224×224 (0.698±0.019), 512×512 (0.664±0.034), and 1024×1024 (0.666±0.024) images. Conversely, the whole-genome method consistently outperformed at 50% and 25% class proportions, especially notable in 512×512 (0.731±0.03 for 50%, 0.797±0.033 for 25%) and 1024×1024 (0.770±0.031 for 50%, 0.790±0.026 for 25%) dimensions, indicating its effectiveness in leveraging comprehensive genomic data. Chromosome-wise and early-integration methods did not produce notable effects, indicating their effectiveness may be limited in this specific context of cultivar-level classification of grapevine samples.

**Figure 6.**
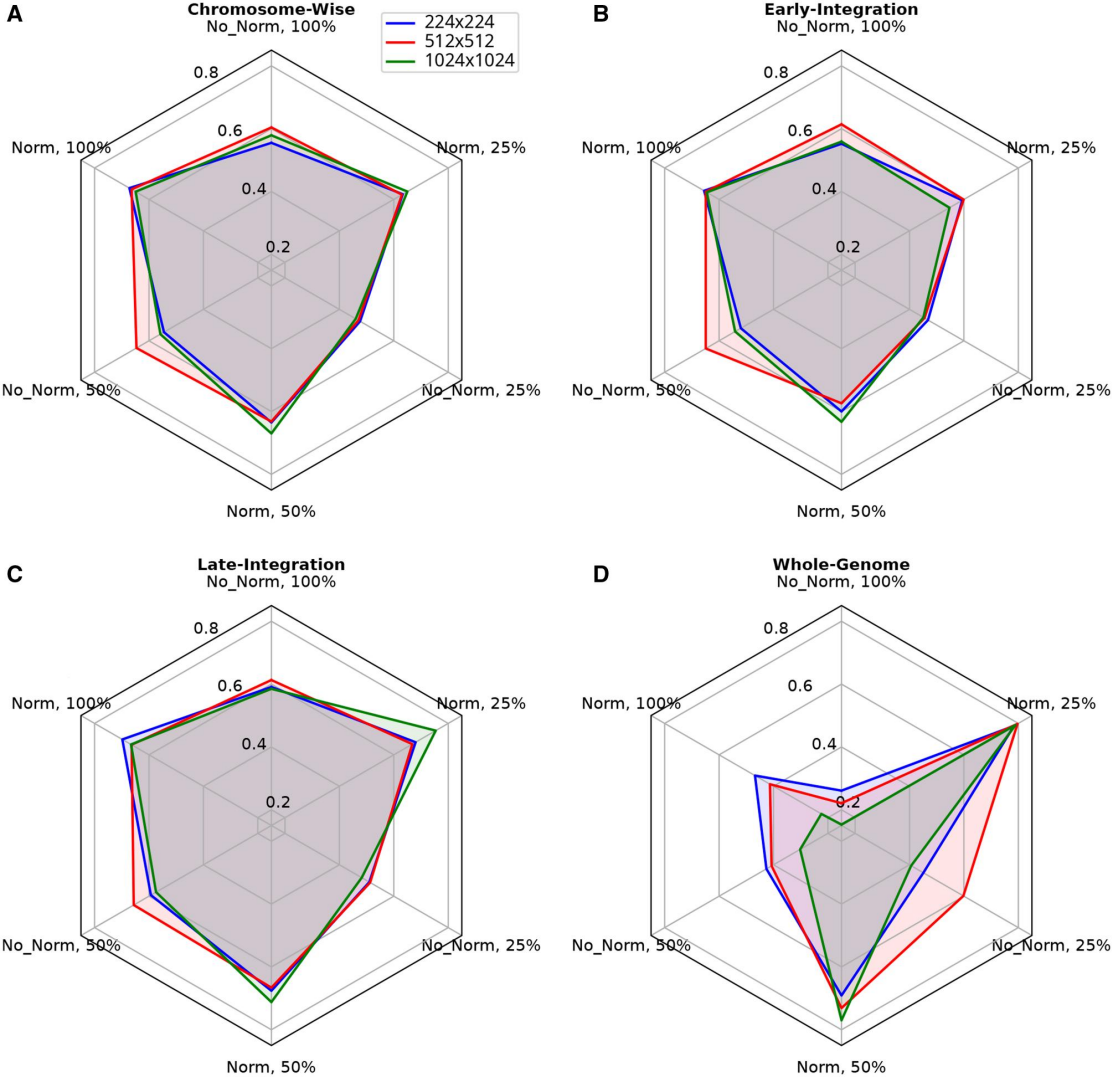
Comparison of CGR resolutions for *Vitis vinifera* cultivar classification. (A) Chromosome-wise method. (B) Early-integration method. (C) Late-integration method. (D) Whole-genome method. Base classifier: ResNet. Mean balanced accuracy on the test set over 20 training runs of each method and configuration is shown.

Figure 6 alternatively illustrates the performance of each resolution in combination with the methods. For 224×224 non-normalized images, late-integration excelled across all class proportions, achieving the highest balanced accuracy at all classes (0.591±0.03), 50% of classes (0.593±0.054), and 25% of classes (0.510±0.025). Conversely, under normalized conditions, the whole-genome method surpassed late-integration at 50% (0.691±0.027) and 25% (0.784±0.03) class proportions, although late-integration led at 100% of classes (0.6989±0.019). In the 512×512 and 1024×1024 dimensions, whole-genome consistently outperformed late-integration at 50% and 25% class proportions, with respective balanced accuracies peaking at (0.797±0.033) for 512×512 and (0.790±0.026) for 1024×1024 images. However, late-integration showed superior balanced accuracy at 100% class proportion across all dimensions. Overall, 512×512 images yielded the most robust performance, especially at 50% and 25% class proportions.

Normalization enhanced classification performance across various dimensions and class proportions, particularly benefiting the whole-genome method by mitigating extreme outliers in CGR generation. The impact of normalization on classification was prominent. In general, normalized images yielded improved performance across different methods and class proportions. This was especially notable with the whole-genome method, where normalization saw increased performance across all class proportions and dimensions. This effect is particularly notable with 224×224 and 1024×1024. The more pronounced effect of normalization on the whole-genome approach can be explained in part by the wholistic nature of the whole-genome method. Given that the whole-genome method captures all chromosomes, normalization may be particularly impactful on extreme outlier pixels during the coordinate calculation step of CGR generation.

Given the total class number of 164 for cultivars, it is clear that the results for cultivar-level classification are far above that of random guess, strongly suggesting that our method has potential as a complementary tool for classifying samples by cultivar. However, given that the results are far below an ideal level of certainty, more work may still be needed for cultivar-level classification.

### 4.5 Base classifier performance comparison

In initial tests, we found that ResNet as base classifier performed the greatest, with ViT performing slightly worse. To further optimize the performance of ViT, we performed a comprehensive test of settings for CGR images with the 224×224 resolution. Table 1 provides a comparison of the top-performing settings of the two base classification models across various experimental conditions, showing the mean balanced accuracy over 20 runs. A comprehensive table can be found in the supplementary data file (Tables S5 and S6, available as supplementary data at *Bioinformatics Advances* online). These include the datasets (species and cultivar), class proportions, normalization procedures, and integration methods (chromosome-wise, early-integration, late-integration, and whole-genome). Similar to the results from ResNet classification, ViT saw the best results with normalized CGR images at lower class size proportion, while late-integration performed the best among the methods. Across both species and cultivar datasets and all parameters, ResNet consistently demonstrated superior performance compared to ViT. Notably, the best-performing results for both models were achieved using 25% class proportion, normalized species data using late-integration, in which ResNet (0.947) outperformed ViT (0.857). For the cultivar datasets, ViT in late-integration showed better results than other three integration strategies. However, the results were notably poorer than for species identification, suggesting that it is more challenging to capture the more subtle differences among cultivars within the *Vitis vinifera* species. Overall, this analysis underscores the robustness and higher accuracy of ResNet across varied datasets and integration methods, suggesting its suitability for classification tasks with CGR images.

**Table 1.** Mean balanced accuracy comparison of base classifiers on 25% class proportion with normalized CGRs at 224 resolution.

Dataset	Method	ViT	ResNet
species	chr-wise	0.796	0.859
species	early-integration	0.623	0.856
species	late-integration	**0.857**	**0.947**
species	whole-genome	0.200	0.600
cultivar	chr-wise	0.194	0.633
cultivar	early-integration	0.070	0.593
cultivar	late-integration	**0.310**	0.680
cultivar	whole-genome	0.048	**0.783**

A bold value indicates the best result for one dataset and model.

### 4.6 Benchmarking

Although there are no existing approaches available for direct comparison with our approaches in the tasks of interest, we developed several methods based on sequence similarity and *k*-mer representation and compared them with the CGR-based approaches for classification of the five grapevine species with the most available samples. The results are shown in Table 2.

**Table 2.** Comparison of CGR-based methods with other methods for grapevine species classification.

Method	Balanced accuracy
Random guess	0.128
*k*-NN (k=1)	0.243
Kmer-db (*k* = 21)	0.780
Kernel SVM (*C* = 2)	0.867
ViT (224, late-integration)	0.857
ResNet (224, late-integration)	0.947
ResNet (1024, late-integration)	**0.990**

The bold value indicates the best result.

First, the *k*-nearest neighbour (*k*-NN) method evaluates sequences based on pairwise similarity scores, calculated as the proportion of matching nucleotides relative to mismatches and gaps in the pseudo-sequences representing the genotype of the variants. For each test sequence, the *k*-nearest sequences from the training set were identified based on the highest similarity scores. The test set includes 57 query samples compared to the training set. The hyperparameter k=1 worked the best among other *k* values in terms of balanced accuracy. This approach had difficulty in identifying matches for each query sequence to the training samples as a result of high similarity and low class distinction. The performance of the CGR models, particularly with ResNet using the late-integration strategy, outperformed *k*-NN, suggesting the ability of CGR-based approaches to leverage deep feature extraction for highly similar sequences.

A *k*-mer-based method was developed to provide a second method for comparison. For this, the Kmer-db tool (Deorowicz *et al.* 2018) was employed to build the *k*-mer database for the same set of pseudo-sequences used to generate the training samples for species in the CGR approach. In the case of Kmer-db, the genome sequences (concatenating individual chromosome sequences) were used for simplicity and increased sequence information. The same set of test samples was used to search the above *k*-mer database, and the species call for each test sample was based on the highest *k*-mer similarity to the training samples in the database. This is actually a *k*-mer-based nearest neighbour method. As shown in Table 2, this method obtained a balanced accuracy of 0.780, much higher than the direct sequence similarity based representation. We explored the values of the hyperparameter *k* in *k*-mer and report that k=21 obtained the best test performance.

Kmer-db does not provide a vector representation for each sample, but instead generates a database to allow the inquiry of a new sample to the database and returns pair-wise similarity values. This functionality enables us to obtain kernel matrices and thus to use kernel methods for species classification. We trained the start-of-the-art kernel support vector machine (SVM) (Scholkopf and Smola 2001) using scikit-learn (Pedregosa *et al.* 2011) on the train-train kernel matrix and tested it using the test-train kernel matrix. Same as in our approach, the classes were weighted to address the class imbalance problem. The hyperparameter *C* was explored and C=2 worked the best. Table 2 shows the mean balanced accuracy over 20 runs. It outperforms the Kmer-db-based nearest neighbour approach but is inferior to ResNet.

Furthermore, we compared the *k*-mer-based approach and kernel SVM with the CGR-based deep learning approaches for classification of cultivar test samples from 39 classes (207 training samples and 53 test samples) and show the results in Table 3. Interestingly, Kmer-db and kernel SVM outperformed ResNet in this task. This is because the class sizes of the cultivars are very small, and most classes have only 1–2 training samples available. ResNet does not have sufficient data to learn in this task, while the Kmer-db method is instance-based without a learning process, and kernel SVM traditionally works well on sparse data. This comparison encourages future work towards hybrid representation and learning approaches to take advantage of the merits of these approaches.

**Table 3.** Comparison of CGR-based methods with other methods for grapevine cultivar classification.

Method	Balanced accuracy
Random guess	0.026
Kmer-db (*k* = 21)	**0.910**
Kernel SVM (*C* = 10)	0.881
ViT (224, late-integration)	0.310
ResNet (512, whole-genome)	0.797
ResNet (1024, whole-genome)	0.790

The bold value indicates the best result.

## 5 Discussion and conclusion

This study investigated the integration of high-throughput genome sequencing, CGR, and deep image models for identification of grapevine species and *Vitis vinifera* cultivars. It is revealed that Robust Scaling effectively normalized data while preserving central values, with normalization generally enhancing results. Optimal CGR image resolutions were identified as 224×224, 512×512, and 1024×1024, with larger resolutions tending to result in notable performance increases for species classification in use with the ResNet18 model. Addressing class imbalances, especially prevalent in *Vitis vinifera*, was crucial, with species-level classification showing marked accuracy improvements in top 25% class samples, particularly with 1024×1024 images (0.990±0.024), while cultivar-level classification did not display similar trends, due to a more balanced class distribution. Late-integration method consistently outperformed others in species-level classification and also showed better results in cultivar-level classification, though the whole-genome method excelled at lower class proportions, suggesting its proficiency in capturing comprehensive genomic patterns. The exhaustive experimentation underscored late-integration and whole-genome as promising approaches in grapevine classification via CGR images.

However, the limitations of greyscale CGR images in capturing intricate patterns indicate potential areas for future research, including exploring advanced normalization techniques. The implementation of other class imbalance mitigation strategies may also enhance model accuracy. Other limitations include the availability of higher-quality data (e.g. better and more homogeneous genome coverage) and sufficiency of sample for minority classes. Improvements may be made through enhancing the quality of pseudo-DNA sequences (representing the genotypes of variants), by using pangenome to increase the variant detection accuracy and by optimizing the parameters to minimize noise in variant calls. Further, seeing the large impact of class size on accuracy, future effort can be made towards acquiring large datasets for training.

Exploration of additional methodological advancements should also be beneficial. In our work, we described the use of four integration methods for model fine-tuning. The development of novel approaches, or hybrid approaches that combine the strengths of different methods or ensemble techniques may lead to increased performance. An example of this may be identifying which chromosome-wise models are most accurate in the task of classification and using that to implement a weighted voting system in which better-performing models have a larger weight in the final classification output. The performance of other modern deep vision models should also be explored within the generic framework for grapevine classifications. The established models and optimal configurations for cultivar classification from this work serve as a stepping stone for future work, particularly for extending into the even more challenging analysis of grapevine clones. Furthermore, once fully tested and validated in grapevine, the established approach can be easily transferred to genetic testing for other crops/species. Importantly, unlike regular images which can be highlighted using heatmaps to visually explain which parts of an image contribute to deep-learning-driven prediction (Selvaraju *et al.* 2017), the CGR-based methods struggle with direct interpretability due to information loss in sequence to image conversion, although it was shown that CGR patterns could reflect unique patterns for specific genes in specific groups of organisms as discussed in length by the original author of the algorithm (Jeffrey 1990). An interesting future work could be the development of explainable grapevine species and cultivar classification models, with which we can identify fragments of sequences or variants contributing to prediction. Additionally, the better results for cultivar classifications with Kmer-db and kernel SVM hints the value of exploring hybrid approaches for classification at species, cultivar, and clone levels. Finally, we are aware and will keep track of the recent development of techniques inspired from natural language processing for DNA sequence modelling with sequence length and tokenization as major challenges. Among them, the maximal input lengths for DNABERT (Ji *et al.* 2021), Nucleotide Transformer (Dalla-Torre *et al.* 2025), and HyenaDNA (Nguyen *et al.* 2023) are 512, 6K, and 1Mbps, respectively, which are far less than the sequence length of a single grapevine chromosome we processed. We attempted to use HyenaDNA to encode partial sequence per chromosome. However, we were unable to configure HyenaDNA by following their instructions.

## Supplementary Material

vbaf193_Supplementary_Data

## Data Availability

The WGS data for a total of 4312 grapevine samples underlying this article are available in the NCBI SRA (https://www.ncbi.nlm.nih.gov/sra) and CNCB GSA (https://ngdc.cncb.ac.cn/gsa) databases, and the full list of accession numbers is available in SampleAccessionList.csv under https://github.com/pliang64/CGR.
