# Supplementary material for: Exploration of chaos game representation and integrative deep learning approaches for whole-genome sequencing-based grapevine genetic testing
Source: Bioinform Adv. 2025 Sep 1;5(1):vbaf193. doi: 10.1093/bioadv/vbaf193 (PMC12449056; doi:10.1093/bioadv/vbaf193)
Supplement: vbaf193_Supplementary_Data [file vbaf193_supplementary_data.pdf]

Bioinformatics Advances, 2025, 1–1
doi: DOI HERE
Advance Access Publication Date: Day Month Year
Paper

Supplementary Data for "Exploration of Chaos Game Representation and Integrative Deep Learning Approaches for Whole-genome Sequencing-Based Grapevine Genetic Testing"

Andrew Vu,[1] Brendan Park,[1] Yifeng Li[1,2,3,*] and Ping Liang[2,3,*]

[1]Department of Computer Science, Brock University, 1812 Sir Isaac Brock Way, St. Catharines, L2S 3A1, Ontario, Canada, [2]Department of Biological Sciences, Brock University, 1812 Sir Isaac Brock Way, St. Catharines, L2S 3A1, Ontario, Canada and [3]Centre for Biotechnology, Brock University, 1812 Sir Isaac Brock Way, St. Catharines, L2S 3A1, Ontario, Canada

[*]To whom correspondence should be addressed. yli2@brocku.ca;pliang@brocku.ca

Abstract

This supplementary data file accompanies the study, titled "Exploration of Chaos Game Representation and Integrative Deep Learning Approaches for Whole-genome Sequencing-Based Grapevine Genetic Testing", and provides the raw data and complete experimental results supporting the main manuscript.

© The Author 2025. Published by Oxford University Press. All rights reserved. For permissions, please e-mail: journals.permissions@oup.com

Table 1. Sample Count by Species for Species Classification.

Species	Training	Validation	Test
Vitis_vinifera	42	7	11
Vitis_hybrid_cultivar	21	4	5
Vitis_cinerea	20	3	6
Vitis_mustangensis	16	3	5
Vitis_arizonica	16	3	4
Vitis_riparia	14	3	4
Vitis_girdiana	13	2	4
Vitis_labrusca	7	2	2
Vitis_rupestris	7	2	2
Vitis_aestivalis	5	1	2
Vitis_amurensis	5	1	2
Vitis_monticola	4	1	2
Vitis_acerifolia	2	1	1
Vitis_davidii	2	1	1
Vitis_labrusca_x_Vitis_vinifera	2	1	1
Vitis_coignetiae	1	1	1
Vitis_romanetii	1	1	1
Vitis_rotundifolia	1	1	1
Vitis_x_doaniana	1	1	1
Total	198	40	59

Table 2. Sample Count by *Vitis vinifera* Cultivar for Cultivar Classification.

Cultivar	Training	Validation	Test
aramon	7	1	2
cabernet_franc	7	2	2
chasselas_blanc	7	2	2
pak	7	2	2
cabernet_sauvignon	6	1	2
cerovica	6	1	2
primitivo	6	1	2
ps	6	1	2
zinfandel	5	1	2
afus_ali	4	1	1
bouschet_petit	4	1	1
khusaine_belyi	4	1	1
kishmish_chernyi	4	1	2
malvasia_bianca_lunga	4	1	2
mj	4	1	1
muscat_a_petits_grains_blancs	4	1	1
muscat_of_alexandria	4	1	1
ps_nn	4	1	1
rkatsiteli	4	1	1
s	4	1	1
sangiovese	4	1	1
trebbiano_toscano	4	1	1
alvarinho	3	1	1
bayan_shirei	3	1	1
chaouch_blanc	3	1	1
feteasca_alba	3	1	1
garnacha_tinta	3	1	1
italia	3	1	1
khindogny	3	1	1
listan_prieto	3	1	1
pak_nn	3	1	1
pinot_noir	3	1	1
solnechnaya_dolina	3	1	1
tannat	3	1	1
verdot_petit	3	1	1
aleatico	2	1	1
black_monukka	2	1	1
chasselas_rose	2	1	1
cinsaut	2	1	1
coarna_alba	2	1	1
csaba_gyoengye	2	1	1
dardagan	2	1	1
dzrali	2	1	1
garganega	2	1	1
graciano	2	1	1
harslevelue	2	1	1
iordan	2	1	1
irsai_oliver	2	1	1
kadarka_kek	2	1	1
katta_kurgan	2	1	1
khamirak	2	1	1
madeleine_angevine	2	1	1
malbec	2	1	1
malvasia_di_sardegna	2	1	1
marawi	2	1	1
misgyuli_kara	2	1	1
mj_nn	2	1	1
monastrell	2	1	1

munage	2	1	1
muscat_hamburg	2	1	1
muscat_ottonel	2	1	1
nebbiolo	2	1	1
negru_virtos	2	1	1
pamid	2	1	1
plechistik	2	1	1
posip_bijeli	2	1	1
pribidrag	2	1	1
red_globe	2	1	1
riesling_weiss	2	1	1
ruby_seedless	2	1	1
rumi_abyad	2	1	1
sauvignon_blanc	2	1	1
schiava_grossa	2	1	1
sultanina	2	1	1
sylvestris_france	2	1	1
syrah	2	1	1
tagobi	2	1	1
taifi_rozovyi	2	1	1
tebrizi	2	1	1
trousseau_noir	2	1	1
tsulukidzis_tetra	2	1	1
voskeat	2	1	1
welschriesling	2	1	1
wild_european	2	1	1
al_burla	1	1	1
alicante_henri_bouschet	1	1	1
aligote	1	1	1
alphonse_lavallee	1	1	1
amud	1	1	1
angur_kalan	1	1	1
aramon_noir	1	1	1
areni_sev	1	1	1
arevik_spitak	1	1	1
asyl_kara	1	1	1
bakator_belyi	1	1	1
berbecel	1	1	1
bical	1	1	1
blaufraenkisch	1	1	1
braghina_rosie	1	1	1
cardinal	1	1	1
carignan_noir	1	1	1
centennial_seedless	1	1	1
chardonnay_blanc	1	1	1
clairette_blanche	1	1	1
colombard	1	1	1
courbu_blanc	1	1	1
crimson_seedless	1	1	1
dodrelyabi	1	1	1
emperor	1	1	1
fantasy_seedless	1	1	1
fernao_pires	1	1	1
feteasca_neagra	1	1	1
feteasca_regala	1	1	1
flame_seedless	1	1	1
furmint	1	1	1
gamay_teinturier_de_bouze	1	1	1

gamay_teinturier_freaux	1	1	1
gold	1	1	1
gorula	1	1	1
grand_noir	1	1	1
gyulyabi_dagestanskii	1	1	1
juhfark	1	1	1
kefessiya	1	1	1
khalili_belyi	1	1	1
kishmish_vatkana	1	1	1
koevidinka	1	1	1
korinthiaki	1	1	1
lambrusco_di_sorbara	1	1	1
longyan	1	1	1
magliocco_dolce	1	1	1
merlot_noir	1	1	1
molar	1	1	1
mskhali	1	1	1
mtsvane_kakhuri	1	1	1
nakhiduri	1	1	1
nazeli	1	1	1
negro_amaro	1	1	1
nero_davola	1	1	1
ninotsminda	1	1	1
palomino_fino	1	1	1
perlette	1	1	1
picolit	1	1	1
pinot_blanc	1	1	1
pinot_gris	1	1	1
polkovnik_izyum	1	1	1
portugieser_blau	1	1	1
rkatsiteli_vardisperi	1	1	1
rufete	1	1	1
saint_laurent	1	1	1
saperavi	1	1	1
semillon	1	1	1
shafei	1	1	1
slankamenka_bela	1	1	1
suntory_blanc	1	1	1
tavkveri	1	1	1
tedotsminda	1	1	1
tempranillo_tinto	1	1	1
terbash	1	1	1
tigvoasa	1	1	1
touriga_nacional	1	1	1
vasarga_belaya	1	1	1
vinhao	1	1	1
vs	1	1	1
zilavka	1	1	1
Total	326	167	175

Table 3. Test Performance (Balanced Accuracy) of *Species* Classification Using ResNet. Mean and standard deviation (STD) over 20 runs for each configuration are shown.

Classes	Norm	Imbal	Method	224	512	1024
100%	No	Very	chr-wise	0.263 (0.023)	0.155 (0.018)	0.223 (0.03)
100%	No	Very	early_integration	0.333 (0.038)	0.222 (0.036)	0.296 (0.054)
100%	No	Very	late_integration	0.293 (0.043)	0.2 (0.027)	0.243 (0.045)
100%	No	Very	whole_genome	0.272 (0.039)	0.23 (0.017)	0.271 (0.054)
100%	No	Mildly	chr-wise	0.344 (0.015)	0.207 (0.027)	0.268 (0.025)
100%	No	Mildly	early_integration	0.352 (0.033)	0.255 (0.025)	0.308 (0.028)
100%	No	Mildly	late_integration	**0.411 (0.033)**	0.221 (0.041)	0.309 (0.038)
100%	Yes	Very	chr-wise	0.320 (0.045)	0.262 (0.029)	0.243 (0.029)
100%	Yes	Very	early_integration	0.326 (0.043)	0.326 (0.032)	0.327 (0.043)
100%	Yes	Very	late_integration	0.319 (0.041)	0.342 (0.044)	0.308 (0.053)
100%	Yes	Very	whole_genome	0.254 (0.046)	0.291 (0.027)	**0.351 (0.045)**
100%	Yes	Mildly	chr-wise	0.337 (0.033)	0.295 (0.015)	0.286 (0.024)
100%	Yes	Mildly	early_integration	0.339 (0.036)	0.328 (0.035)	0.339 (0.04)
100%	Yes	Mildly	late_integration	0.339 (0.029)	**0.346 (0.038)**	0.338 (0.042)
50%	No	Very	chr-wise	0.454 (0.026)	0.336 (0.047)	0.459 (0.053)
50%	No	Very	early_integration	0.516 (0.047)	0.416 (0.051)	0.561 (0.078)
50%	No	Very	late_integration	0.475 (0.042)	0.454 (0.083)	0.548 (0.052)
50%	No	Very	whole_genome	0.437 (0.091)	0.431 (0.045)	0.499 (0.079)
50%	No	Mildly	chr-wise	0.545 (0.022)	0.447 (0.03)	0.539 (0.046)
50%	No	Mildly	early_integration	0.549 (0.057)	0.437 (0.058)	0.558 (0.059)
50%	No	Mildly	late_integration	0.591 (0.047)	0.48 (0.037)	0.597 (0.048)
50%	Yes	Very	chr-wise	0.443 (0.045)	0.531 (0.03)	0.505 (0.03)
50%	Yes	Very	early_integration	0.479 (0.053)	0.659 (0.059)	0.585 (0.052)
50%	Yes	Very	late_integration	0.525 (0.067)	0.675 (0.041)	0.663 (0.033)
50%	Yes	Very	whole_genome	0.485 (0.079)	0.372 (0.066)	0.550 (0.051)
50%	Yes	Mildly	chr-wise	0.631 (0.074)	0.64 (0.037)	0.629 (0.042)
50%	Yes	Mildly	early_integration	0.609 (0.08)	0.609 (0.058)	0.599 (0.058)
50%	Yes	Mildly	late_integration	**0.687 (0.065)**	**0.696 (0.058)**	**0.694 (0.059)**
25%	No	Very	chr-wise	0.488 (0.055)	0.505 (0.048)	0.479 (0.042)
25%	No	Very	early_integration	0.552 (0.041)	0.541 (0.084)	0.541 (0.037)
25%	No	Very	late_integration	0.552 (0.09)	0.496 (0.101)	0.512 (0.078)
25%	No	Very	whole_genome	0.640 (0.142)	0.504 (0.076)	0.800 (0.093)
25%	No	Mildly	chr-wise	0.881 (0.019)	0.848 (0.025)	0.783 (0.03)
25%	No	Mildly	early_integration	0.817 (0.057)	0.853 (0.035)	0.733 (0.061)
25%	No	Mildly	late_integration	0.920 (0.027)	0.88 (0.035)	0.877 (0.062)
25%	Yes	Very	chr-wise	0.622 (0.05)	0.48 (0.046)	0.447 (0.016)
25%	Yes	Very	early_integration	0.619 (0.047)	0.512 (0.065)	0.444 (0.049)
25%	Yes	Very	late_integration	0.569 (0.077)	0.471 (0.152)	0.474 (0.044)
25%	Yes	Very	whole_genome	0.600 (0)	0.532 (0.063)	0.775 (0.044)
25%	Yes	Mildly	chr-wise	0.860 (0.092)	0.815 (0.023)	0.857 (0.037)
25%	Yes	Mildly	early_integration	0.857 (0.073)	0.82 (0.062)	0.890 (0.033)
25%	Yes	Mildly	late_integration	**0.947 (0.027)**	**0.88 (0.041)**	**0.990 (0.024)**

Table 4. Test Performance (Balanced Accuracy) of *Cultivar* Classification Using ResNet. Mean and standard deviation (STD) over 20 runs for each configuration are shown.

Num_Classes	Norm	Method	224	512	1024
100%	No	chr-wise	0.555 (0.021)	0.604 (0.015)	0.579 (0.015)
100%	No	early_integration	0.552 (0.021)	0.614 (0.02)	0.559 (0.017)
100%	No	late_integration	0.591 (0.03)	0.613 (0.013)	0.585 (0.024)
100%	No	whole_genome	0.261 (0.022)	0.221 (0.031)	0.153 (0.021)
100%	Yes	chr-wise	0.672 (0.015)	**0.664 (0.019)**	0.649 (0.017)
100%	Yes	early_integration	0.655 (0.013)	0.649 (0.018)	0.645 (0.018)
100%	Yes	late_integration	**0.698 (0.019)**	**0.664 (0.034)**	**0.666 (0.024)**
100%	Yes	whole_genome	0.468 (0.047)	0.413 (0.069)	0.224 (0.099)
50%	No	chr-wise	0.545 (0.039)	0.646 (0.03)	0.558 (0.012)
50%	No	early_integration	0.520 (0.023)	0.649 (0.029)	0.541 (0.018)
50%	No	late_integration	0.593 (0.054)	0.656 (0.031)	0.574 (0.014)
50%	No	whole_genome	0.426 (0.028)	0.407 (0.055)	0.302 (0.025)
50%	Yes	chr-wise	0.635 (0.017)	0.632 (0.025)	0.670 (0.027)
50%	Yes	early_integration	0.600 (0.025)	0.574 (0.038)	0.633 (0.047)
50%	Yes	late_integration	0.676 (0.023)	0.666 (0.024)	0.712 (0.049)
50%	Yes	whole_genome	**0.691 (0.027)**	**0.731 (0.03)**	**0.770 (0.031)**
25%	No	chr-wise	0.476 (0.015)	0.470 (0.029)	0.460 (0.012)
25%	No	early_integration	0.468 (0.033)	0.455 (0.04)	0.451 (0.02)
25%	No	late_integration	0.510 (0.025)	0.513 (0.061)	0.482 (0.016)
25%	No	whole_genome	0.451 (0.032)	0.598 (0.029)	0.406 (0.052)
25%	Yes	chr-wise	0.633 (0.033)	0.630 (0.028)	0.650 (0.038)
25%	Yes	early_integration	0.594 (0.039)	0.599 (0.039)	0.547 (0.076)
25%	Yes	late_integration	0.680 (0.027)	0.668 (0.018)	0.754 (0.086)
25%	Yes	whole_genome	**0.784 (0.03)**	**0.797 (0.033)**	**0.790 (0.026)**

Table 5. Comparison of Base Classifiers (ViT and ResNet) for *Species* Classification on Test Data in Terms of Mean Balanced Accuracy.

Dataset	Num_Classes	Norm	Balance	Method	ViT	ResNet
species	100%	No	Very	chr-wise	0.177	0.263
species	100%	No	Very	early-integration	0.190	0.333
species	100%	No	Very	late-integration	0.205	0.293
species	100%	No	Very	whole-genome	0.169	0.272
species	100%	No	Mildly	chr-wise	0.162	0.344
species	100%	No	Mildly	early-integration	0.163	0.352
species	100%	No	Mildly	late-integration	0.190	**0.411**
species	100%	Yes	Very	chr-wise	0.210	0.320
species	100%	Yes	Very	early-integration	0.207	0.326
species	100%	Yes	Very	late-integration	0.242	0.319
species	100%	Yes	Very	whole-genome	0.056	0.254
species	100%	Yes	Mildly	chr-wise	0.212	0.337
species	100%	Yes	Mildly	early-integration	0.194	0.339
species	100%	Yes	Mildly	late-integration	**0.261**	0.339
species	50%	No	Very	chr-wise	0.347	0.454
species	50%	No	Very	early-integration	0.354	0.516
species	50%	No	Very	late-integration	0.393	0.475
species	50%	No	Very	whole-genome	0.288	0.437
species	50%	No	Mildly	chr-wise	0.227	0.545
species	50%	No	Mildly	early-integration	0.261	0.549
species	50%	No	Mildly	late-integration	0.296	0.591
species	50%	Yes	Very	chr-wise	0.481	0.443
species	50%	Yes	Very	early-integration	0.368	0.479
species	50%	Yes	Very	late-integration	0.514	0.525
species	50%	Yes	Very	whole-genome	0.100	0.485
species	50%	Yes	Mildly	chr-wise	0.471	0.631
species	50%	Yes	Mildly	early-integration	0.400	0.609
species	50%	Yes	Mildly	late-integration	**0.528**	**0.687**
species	25%	No	Very	chr-wise	0.508	0.488
species	25%	No	Very	early-integration	0.515	0.552
species	25%	No	Very	late-integration	0.536	0.552
species	25%	No	Very	whole-genome	0.429	0.640
species	25%	No	Mildly	chr-wise	0.413	0.881
species	25%	No	Mildly	early-integration	0.403	0.817
species	25%	No	Mildly	late-integration	0.550	0.920
species	25%	Yes	Very	chr-wise	0.777	0.622
species	25%	Yes	Very	early-integration	0.591	0.619
species	25%	Yes	Very	late-integration	0.849	0.569
species	25%	Yes	Very	whole-genome	0.200	0.600
species	25%	Yes	Mildly	chr-wise	0.797	0.860
species	25%	Yes	Mildly	early-integration	0.623	0.857
species	25%	Yes	Mildly	late-integration	**0.857**	**0.947**

Table 6. Comparison of Base Classifiers (ViT and ResNet) for *Cultivar* Classification on Test Data in Terms of Mean Balanced Accuracy.

Dataset	Num_Classes	Norm	Method	ViT	ResNet
cultivar	100%	No	chr-wise	0.070	0.555
cultivar	100%	No	early-integration	0.035	0.552
cultivar	100%	No	late-integration	0.093	0.591
cultivar	100%	No	whole-genome	0.065	0.261
cultivar	100%	Yes	chr-wise	0.124	0.672
cultivar	100%	Yes	early-integration	0.020	0.655
cultivar	100%	Yes	late-integration	**0.141**	**0.698**
cultivar	100%	Yes	whole-genome	0.022	0.468
cultivar	50%	No	chr-wise	0.113	0.545
cultivar	50%	No	early-integration	0.043	0.520
cultivar	50%	No	late-integration	0.120	0.593
cultivar	50%	No	whole-genome	0.128	0.426
cultivar	50%	Yes	chr-wise	0.127	0.635
cultivar	50%	Yes	early-integration	0.045	0.600
cultivar	50%	Yes	late-integration	**0.270**	0.676
cultivar	50%	Yes	whole-genome	0.034	**0.691**
cultivar	25%	No	chr-wise	0.133	0.476
cultivar	25%	No	early-integration	0.041	0.468
cultivar	25%	No	late-integration	0.186	0.510
cultivar	25%	No	whole-genome	0.161	0.451
cultivar	25%	Yes	chr-wise	0.195	0.633
cultivar	25%	Yes	early-integration	0.070	0.594
cultivar	25%	Yes	late-integration	**0.310**	0.680
cultivar	25%	Yes	whole-genome	0.048	**0.784**
